# The neoepitope landscape of breast cancer: implications for immunotherapy

**DOI:** 10.1186/s12885-019-5402-1

**Published:** 2019-03-04

**Authors:** Pooja Narang, Meixuan Chen, Amit A. Sharma, Karen S. Anderson, Melissa A. Wilson

**Affiliations:** 1 School of Life Sciences, Arizona State University, PO Box 874501, Tempe, AZ 85287-4501 USA; 2 Center for Personalized Diagnostics, The Biodesign Institute, Arizona State University, Tempe, AZ USA; 3 Center for Evolution and Medicine, Arizona State University, Tempe, AZ USA

**Keywords:** Breast cancer, Neoepitope prediction, Mutation burden, Immunotherapy, TNBC, Epitopes

## Abstract

**Background:**

Cancer immunotherapy with immune checkpoint blockade (CKB) is now standard of care for multiple cancers. The clinical response to CKB is associated with T cell immunity targeting cancer-induced mutations that generate novel HLA-binding epitopes (neoepitopes).

**Methods:**

Here, we developed a rapid bioinformatics pipeline and filtering strategy, EpitopeHunter, to identify and prioritize clinically relevant neoepitopes from the landscape of somatic mutations. We used the pipeline to determine the frequency of neoepitopes from the TCGA dataset of invasive breast cancers. We predicted HLA class I-binding neoepitopes for 870 breast cancer samples and filtered the neoepitopes based on tumor transcript abundance.

**Results:**

We found that the total mutational burden (TMB) was highest for triple-negative breast cancer, TNBC, (median = 63 mutations, range: 2–765); followed by HER-2(+) (median = 39 mutations, range: 1–1206); and lowest for ER/PR(+)HER-2(−) (median = 32 mutations, range: 1–2860). 40% of the nonsynonymous mutations led to the generation of predicted neoepitopes. The neoepitope load (NEL) is highly correlated with the mutational burden (R^2^ = 0.86).

**Conclusions:**

Only half (51%) of the predicted neoepitopes are expressed at the RNA level (FPKM≥2), indicating the importance of assessing whether neoepitopes are transcribed. However, of all patients, 93% have at least one expressed predicted neoepitope, indicating that most breast cancer patients have the potential for neo-epitope targeted immunotherapy.

**Electronic supplementary material:**

The online version of this article (10.1186/s12885-019-5402-1) contains supplementary material, which is available to authorized users.

## Background

Immune evasion is a hallmark of cancer [1]. Immune checkpoint pathways contribute to cancer-induced immunosuppression, and immune checkpoint blockade (CKB) therapy has been successful in multiple cancers [2, 3]. Blockade of the immune regulatory molecules PD-1/PD-L1 and CTLA-4 reactivates T cell immunity and overcomes underlying T cell exhaustion [4, 5]. Sustained clinical responses and improved survival have been observed in the treatment arms of checkpoint blockade clinical trials, especially in tumors with high mutational burdens, such as melanoma and non-small cell lung cancer (NSCLC) [5–7]. Immune CKB is now FDA proved in melanoma [8], NSCLC [9], head and neck cancer [9] and bladder cancer [10], with ongoing clinical trials in multiple other cancer types.

Even though checkpoint blockade has achieved significant clinical success, it is not universally successful across patients [6, 10, 11]. There are two predictive biomarkers of response to CKB therapies. First, expression of PD-L1 in tumor samples correlates with checkpoint blockade response [11, 12]. Second, there is a strong association between the total mutational burden and clinical response [13–15]. In colorectal cancer, the objective response rate is 40% in tumors with defects in mismatch repair, which have mutational burdens 10–100 times higher than tumors with functional mismatch repair [16].

Neoepitopes are targets for vaccine development [17–19] and adoptive T cell therapy [20]. Neoepitopes occur, in part, as a result of unique somatic mutations in tumor cells, and are predicted to occur in tumors harboring more than 1 somatic mutation per Mb [21]. Mutation-specific CD8+ T cells have been identified in patients who respond to checkpoint blockade [15, 22]**.** The combination of checkpoint blockade with a patient-specific neoepitope vaccines are being developed [23].

Breast cancer typically harbors lower mutational loads than melanoma and NSCLC, averaging one mutation per Mb, but the mutational burden varies both within and across breast cancer subtypes [24]. The most frequently mutated genes are TP53 and PIK3CA [24]. Mutation-specific tumor-infiltrating T cells have been identified in metastases derived from breast cancer patients, and significant tumor responses have been observed after adoptive T cell transfer [25]. High levels of tumor infiltrating lymphocytes are correlated with improved clinical prognosis [26]. Two clinical trials of PD-1/PD-L1 inhibitors in advanced triple negative breast cancer (TNBC) have been reported, highlighting the clinical importance of immunotherapy in breast cancer. First, in the phase Ib KEYNOTE-012 trial of pembrolizumab, a 19% of overall response rate was observed, and, in the phase Ia trial of atezolizumab, a 24% overall response rate was observed [27, 28]. Identification of neoepitopes in breast cancer is essential for monitoring therapies and to generated personalized vaccines.

Next generation sequencing combined with high performance computing has resulted in the exploration and prediction of neoepitopes in multiple cancers [23, 29–31]. The general approach includes HLA-typing and neoepitope prediction from whole exome sequence data [32]. There are also large databases for neoepitope prediction using a combination of somatic mutations and HLA-types; the oldest is SYFPEITHI [33], while the Immune Epitope Database, IEDB [34], is most widely used [32]. A few of the publically available pipelines used NetMHC [35] for epitope prediction like pVAC-seq [36] and INTEGRATE-neo [37]. Prediction methods have been applied to identify neoepitopes in melanoma, NSCLC, and chronic lymphocytic leukemia [14, 19, 23]. In a study of murine melanoma cells, B16F10, 50 validated mutations out of 962 non-synonymous point mutations were tested in vivo, and one-third elicited an immunogenic response [29]. A meta-analysis focused on neoepitopes generated exclusively by missense mutations reported between 1 to 147 immunogenic mutations per patient across 181 patients with different cancer types [38]. However, the neoepitope landscape of breast cancer has not yet been fully explored.

**Fig. 1:**
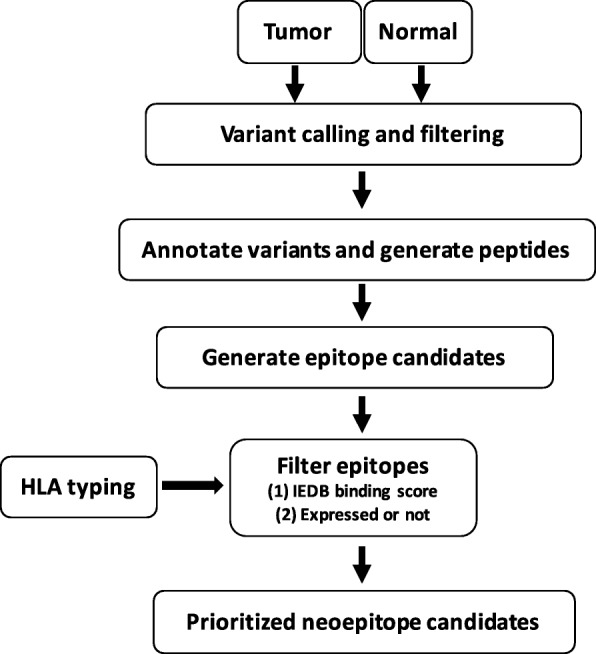
EpitopeHunter: Pipeline to identify clinically relevant neoepitopes. Proposed pipeline to generate and identify clinically relevant neoepitopes from the landscape of somatic mutations from tumor and normal exome sequencing data

In this study, we evaluate the landscape of neoepitope burden across breast cancers included in The Cancer Genome Atlas (TCGA) [39]. We develop an efficient and publicly available prediction pipeline, EpitopeHunter (Fig. 1), to identify and prioritize clinically relevant mutations from the landscape of somatic mutations from tumor and normal exome data, patient-specific HLA-types, and neoepitope expression. Our prediction strategy employs the IEDB [34] binding affinity prediction algorithm, which dynamically selects the best possible method based on the predictive performance of several prediction methods [40–43], for a given MHC molecule. Other publicly available pipelines [36, 37], on the other hand, use only one of the binding affinity prediction method for MHC molecules. We applied our pipeline to 870 breast cancer samples from TCGA [39]. Both somatic mutational burden and neoepitope load varies widely across tumors in breast cancer sub-types, and are highly correlated. We find that half of the predicted neoepitopes are expressed at the RNA level (FPKM≥2). Despite this, 93.5% of the patients have at least one expressed neoepitope that may serve as a candidate for targeted immunotherapy.

## Methods

### Breast cancer samples

The controlled access sequence data from The Cancer Genome Atlas (TCGA) was obtained for all submitted breast cancer samples via the genomics data commons (GDC) data portal with dbGap approval to Dr. Wilson Sayres (https://portal.gdc.cancer.gov; dbGap approval #46688).

### Calling cancer specific variants

Tumor and germline whole exome sequencing BAM files were obtained for each patient, aligned to the GRCh37 human reference genome. We used this version of the genome to compare with the TCGA RNAseq calling and annotation available at the time, for which all samples were aligned to hg19/GRCh37. Samtools version 1.3.1 [44] was used to convert the aligned bam files to the pileup format. High confidence SNPs and indels were retrieved using VarScan2 version 2.3.9 [45] with minimum coverage of 10, minimum variant allele frequency of 0.08 and somatic *p* value of 0.05. Additionally, we removed somatic mutations from the high confidence SNPs that fell within 1 bp of an indel position, which are likely false positives due to alignment errors.

### Variant annotation and neoepitope generation

We annotated mutations identified in the somatic variant calling using the Variant Effect Predictor tool [46] with ensemble transcripts annotated for the hg19 reference genome. For each non-synonymous amino acid, we generated all possible peptides including the mutated amino acid at every position in a sequence with total lengths of 8, 9, 10, 11 amino acids (called –mers) using the utility generate fasta from pvacseq [36]. That is, all possible 8-mers where the mutated amino acid is at the first position, then the second, third, and so on in a sliding window fashion. We also extracted the corresponding non-mutated reference sequence for each potential neoepitope. Thus, for every mutated amino acid we generated 38 possible neoepitopes.

### HLA typing for each patient

We used POLYSOLVER (POLYmorphic loci reSOLVER) [47] to infer the HLA type of each patient using the germline whole exome sequencing data. This method employs a Bayesian classifier and selects and aligns putative HLA reads to an imputed library of full-length HLA alleles. We analyzed three major MHC class I genes (HLA-A, −B, −C) for HLA typing.

### Predicting class I binding epitopes

To find neoepitopes predicted to bind to the patient-specific HLA alleles, we used the consensus prediction method from the Immune Epitope Database (IEDB) [34]. We began by matching the 4-dight HLA type of the patient to the HLA alleles in the IEDB database. If the matching HLA type of the patient did not exist in the current IEDB list, we identified the closest allele by keeping the first two digits same and searching for the best available match for the third and fourth digit. For each combination of HLA allele and peptides for each nonsynonymous amino acid (using those generated as 8, 9, 10, and 11-mers above), Epitopehunter selects the epitopes with lowest IEDB score. Thus, for each allele and mutant amino acid combination, it retains only one epitope. To get all high affinity binding neoepitopes with the patient-specific MHC class I molecules, we filtered epitopes with a binding affinity less than or equal to 500 nM, and for the current study, we call these potential binding neoepitopes.

### RNAseq expression filtering

We obtained the gene level FPKM (Fragments Per Kilobase of transcript per Million mapped reads) values from the GDC portal for tumor samples with matched transcriptome data (https://portal.gdc.cancer.gov). Neoantigens were selected if the gene in which the neo-antigen appears was expressed in that patient’s tumor. Based on our previous analysis of FPKM thresholds [48], a gene was considered to be expressed using a cut-off of FPKM≥2. We also evaluated a more stringent cut-off of FPKM≥5.

### Statistical analysis

All statistical comparisons and correlations were performed using an unpaired *t-*test and variation among and between groups was calculated using ANOVA (GraphPad Prism 6). The clinical data were described by the percentage, Kaplan-Meier method for calculation of survival, Log-Rank method for the univariate factor analysis (GraphPad Prism 6). *P*-values≤0.05 was considered significant. Significance testing of data from the three subtypes was performed using Wilcox rank sum test with *** *P* < 0.001 ** *P* < 0.01.

### Molecular subtyping of breast cancer samples

To assign samples to molecular subtypes of breast cancer, we used the clinical calls of biomarkers using immunohistochemistry (IHC) status available for TCGA data (https://cancergenome.nih.gov). We subdivided the clinical samples into three categories based on the immunohistochemical expression of the estrogen receptor (ER), progesterone receptor (PR) and the HER-2 receptor, into three types: 1) ER/PR(+)HER-2(−), those with ER and/or PR(+), HER-2(−); 2) HER-2(+), regardless of ER/PR status; and, 3) TNBC, triple negative breast cancer negative for all three ER, PR, and HER-2. The total numbers of TCGA samples and those used in this study for each subtype are reported in Additional file 1: Table S1.

## Results

### EpitopeHunter pipleline

The EpitopeHunter pipeline (Fig. 1) is broadly divided into the following steps: (1) variant calling and filtering; (2) variant annotation, peptide generation and generating a list of predicted neoepitopes; (3) HLA typing; and, (4) filtering epitope candidates based on IEDB binding score and epitope expression. The first step calls cancer specific mutations as described in the methods section. The user can use any cancer specific variant calling strategy and submit a variant file in vcf format (variant call format) for the next step. Next, we conduct variant annotation and generate a list of neoepitopes from nonsynonymous variants. This step will generate a total of 38 possible neoepitopes of lengths ranging from 8 to 11 amino acids for every non-synonymous cancer-specific variant. HLA typing can be performed using any of the HLA calling programs available for MHC class I; here we use Polysolver. Next, we predict which neoepitopes have a high likelihood to bind to the patient specific HLA alleles using IEDB and filter the high-affinity binding alleles on the basis of RNAseq expression (Methods). The final output file is a list of filtered/selected neoepitopes with binding scores for both WT and mutant epitopes.

### Patient characteristics suggest a range of primary breast cancer samples

A total of 870 breast cancer patient samples from TCGA were included in our study; characteristics of the patients are summarized in Additional file 2: Table S2. The ER/PR(+)HER-2(−) was the most common subtype (72.4%) in this study, following by the HER-2(+) subtype (16.2%) and TNBC (11.4%). A higher proportion of patients with the TNBC subtype were under 50 years old (37%) compared to non-TNBC (25–26.5%). The majority of patients had early stage tumors; 83.4–88% of patients had a primary tumor size of T1-T2 across subtypes. Lymph node status was negative in less than half of patients with ER/PR(+)HER-2(−) subtype (44.6%) and patients with the HER-2(+) subtype (41.8%), but 68% of patients with the TNBC subtype had a negative lymph node status. Most of the patients included in our analysis were diagnosed with stage I/II breast cancer, with only 14–28.4% of patients were stage III-IV.

### Confirmation of frequently mutated genes in subtypes of breast cancer

We identified the somatic mutational landscape across the three subtypes of breast cancer defined here (Additional file 3: Figure S1). For ER/PR(+)HER-2(−) cases, *PIK3CA* was mutated in 33% of the samples, *TP53* was mutated in 13% of the samples, and *TTN* was mutated in 9% of the samples. In HER-2(+) cases, *PIK3CA* was mutated in 33% of the cases, *TP53* was mutated in 24% of the cases, and TTN was mutated in 10% of cases. The most frequently mutated genes in TNBC cases are *TP53* (present in 40% of samples), *TTN* (present in 17% of samples) and *FAT3* (present in 10% of samples). Mutations called by the current protocol are also comparable with those called previously and available via cBioportal (Additional file 4: Figure S2). The current protocol appears to be more conservative, limiting potential non-synonymous mutations; we have a uniform variant calling protocol to call mutations with high confidence. The total number of nonsynonymous mutations are comparable to reports using previous methods in most cases (Additional file 4: Figure S2), and we chose to err on the side of being conservative with our mutation calling, to limit the number of false positive neoepitopes.

### Mutational burden in subtypes of breast cancer

**Fig. 2:**
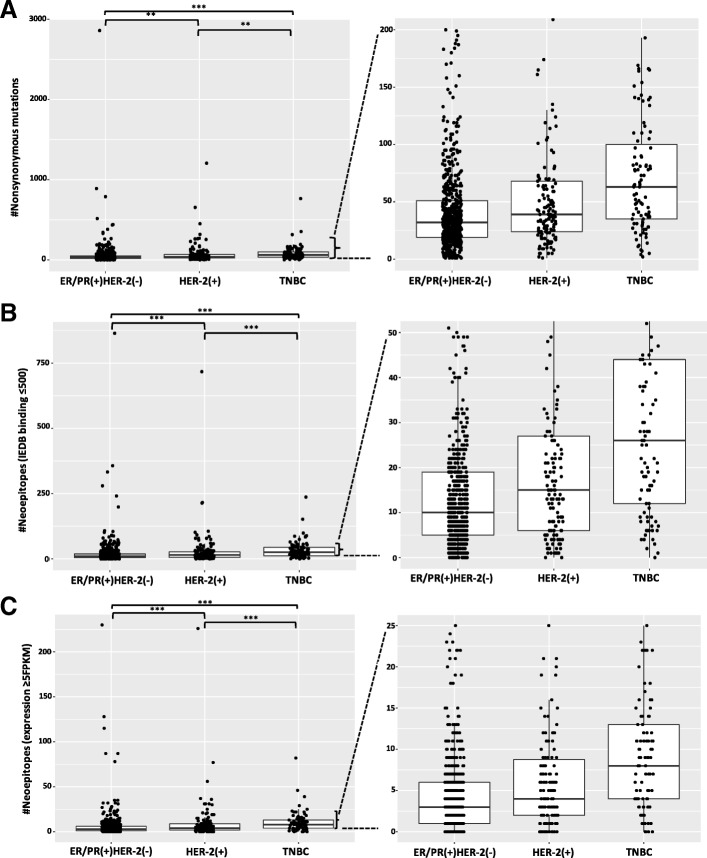
Mutational burden, potential neoepitopes, and expressed neoepitopes in breast cancer determined using Epitopehunter. (**a**) The mutational load is highest in triple negative breast cancer (TNBC), followed by the HER-2(+) breast cancer subtype, and least for the ER/PR(+)HER-2(−) subtype. The median and range of non-synoymous mutations per cancer type are: 32 (1–2860) in ER/PR(+)HER-2(−), 39 (1–1206) in HER-2(+) and 63 (2–765) in TNBC. The number of samples in each subtype are: 630 (ER/PR(+)HER-2(−)), 141 (HER-2(+)), 99 (TNBC). (**b**) The range of potential binding neoepitopes (IEDB score ≤ 500 nM) is highest for the TNBC subtype, followed by HER-2(+); and lowest for the ER/PR(+)HER-2(−) subtype. The median and range of high affinity binding neoepitopes are as follows: 10 (0–864) in ER/PR(+)HER-2(−), 15 (0–717) in HER-2(+) and 26 (0–237) in TNBC. The number of samples in each subtype are: 586 (ER/PR(+)HER-2(−)), 138 (HER-2(+)), 93 (TNBC). (**c**) The median and range of predicted neoepitope with expression (FPKM ≥5) across breast cancer subtypes are: 3 (0–230) in ER/PR(+)HER-2(−), 4 (0–226) in HER-2(+) and 8(0–82) in TNBC. The number of samples in each case are: 583(ER/PR(+)HER-2(−)), 138(HER-2(+)), 92(TNBC). Significant differences between subtypes of cancer are computed pairwise for each breast cancer subtype using a Wilcox rank sum test, *** *P* < 0.001 ** *P* < 0.01

Mutational burden has a large range both within and between breast cancer subtypes (Fig. 2a). Overall, we observed the highest median nonsynonymous mutational burden among samples from patients with TNBC (median = 63 nonsynonymous mutations, range: 2–765); followed by HER-2(+) (median = 39 nonsynonymous mutations, range: 1–1206); and the lowest median mutational burden for samples from patients with ER/PR(+)HER-2(−) tumors (median = 32 nonsynonymous mutations, range: 1–2860). The mutational burden overlaps significantly across breast cancer subtypes, with extreme outliers on either end of the range (Fig. 2a).

### Range of generated neoepitopes in subtypes of breast cancer

**Fig. 3:**
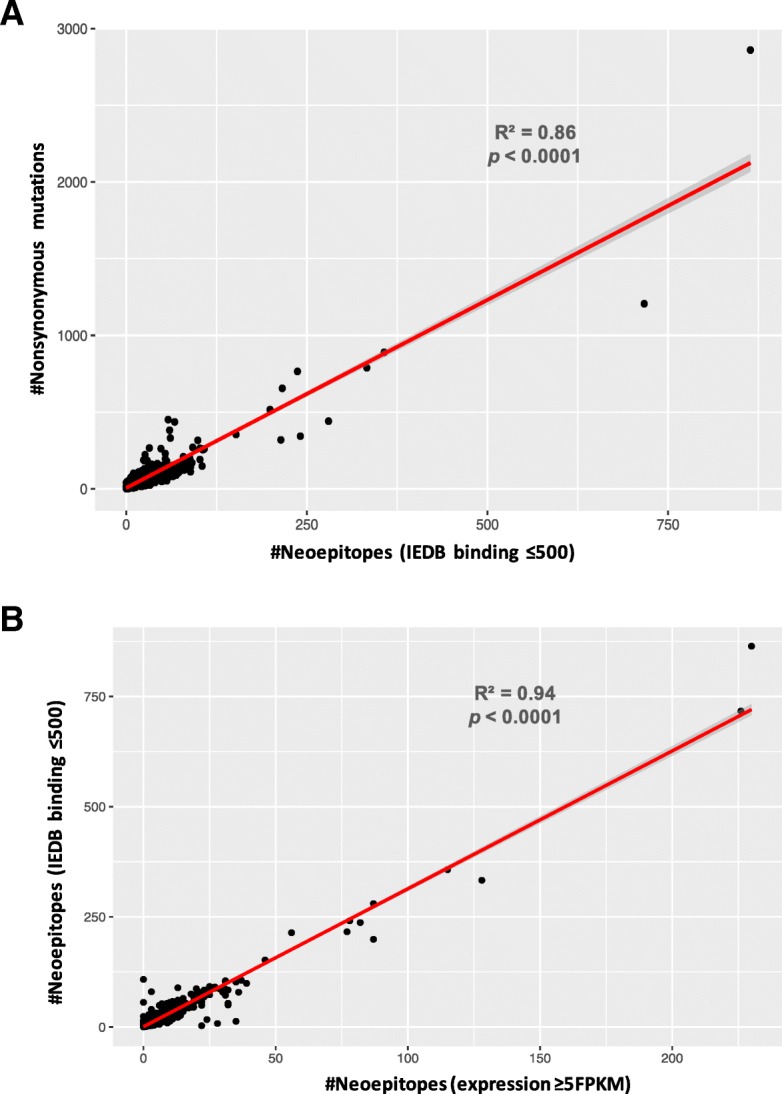
Correlations between predicted neoepitopes, mutational burden, and expressed neoepitopes. (**a**) The number of predicted binding neoepitopes (IEDB score ≤ 500 nM) is highly correlated (R^2^ = 0.86, *p* < 0.0001) with the number of nonsynonymous mutations across all breast cancers. (**b**) The number of predicted binding neoepitopes (IEDB score ≤ 500 nM) is highly correlated (R^2^ = 0.94, *p* < 0.0001) with the number of expressed neoepitopes (FPKM≥5) in all breast cancers. A fitted line from a linear regression is shown in red, with 95% CI levels shown in the grey shaded areas

For each patient sample, we generated all possible neoepitopes from nonsynonymous mutations called in each tumor sample relative to the patient-specific germline (Methods). We selected the potential binding neoepitopes (neoepitope load) as those with ≤500 nM binding affinity to the set of patient-specific HLA class I alleles (Methods). Across tumors subtypes, ~ 37% of the nonsynonymous mutations in the ER/PR(+)HER-2(−) subset, ~ 41% of nonsynonymous mutations in the HER-2(+) subset and ~ 43% of the nonsynonymous mutations in the TNBC subset had a binding affinity ≤500 nM and were called as potential binding neoepitopes. Following the trend for the median mutational burden, the number of potential binding neoepitopes is highest for TNBC (median = 26, range:0–237), followed by HER-2(+) (median = 15, range:0–717); and is lowest for ER/PR(+)HER-2(−) (median = 10, range:0–864; Fig. 2b). The neoepitope load is highly correlated with the mutational burden in all the breast cancer samples considered together (R^2^ = 0.86, *p* < 0.001; Fig. 3a), or when broken up by subtypes (Additional file 5: Figure S3): ER/PR(+)HER-2(−) (R^2^ = 0.90, *p* < 0.001), HER-2(+) (R^2^ = 0.86, *p* < 0.001), and TNBC (R^2^ = 0.84, *p* < 0.001). The ratio of somatic mutational burden to predicted neoepitope load for the entire breast cancer dataset is 2.5:1 (Fig. 3a), similar to published values [49, 50].

We tested for the binding of all possible neoepitopes in sliding windows around the mutation of size 8 amino acids (8-mer), 9-mer, 10-mer, and 11-mer, with the patient-specific HLA (Methods), and found potential binding neoepitopes were largely skewed towards 9-mers. In particular, we find that the highest affinity binding epitope for any mutation is the 9-mer 57% of the time, in contrast to the 10-mers (33%), 11-mers (6%) and 8-mers (4%; Additional file 6: Figure S4).

### Filtering neoepitopes on the basis of expression

We sought to identify neoepitopes that are expressed, and thus likely to elicit a response with immunotherapy. For this, we measured the expression of potential binding neoepitopes using available RNAseq data. We included neoepitopes as expressed if the gene in which the mutation occurred was expressed with FPKM≥2 (Rupp et al., 2017; Additional file 7: Figure S5). Additionally, we also tested a more stringent cutoff of FPKM≥5 (Fig. 2c). The number of expressed neoepitopes (cutoff of FPKM≥5; Fig. 2c) is highest for TNBC (median = 8 epitopes, range:0–82), followed by HER-2(+) (median = 4 epitopes, range:0–226), and lowest for ER/PR(+)HER-2(−) (median = 3 epitopes, range:0–230) subtype. As we expected, the neoepitope burden across all breast cancers is highly correlated with the number of expressed neoepitopes (R^2^ = 0.94, *p* < 0.001) (Fig. 3b), as well as each of the three subtypes individually (Additional file 8: Figure S6): TNBC (R^2^ = 0.93, *p* < 0.001), HER-2(+) (R^2^ = 0.99, *p* < 0.001), ER/PR(+)HER-2(−) (R^2^ = 0.95, *p* < 0.001).

**Fig. 4:**
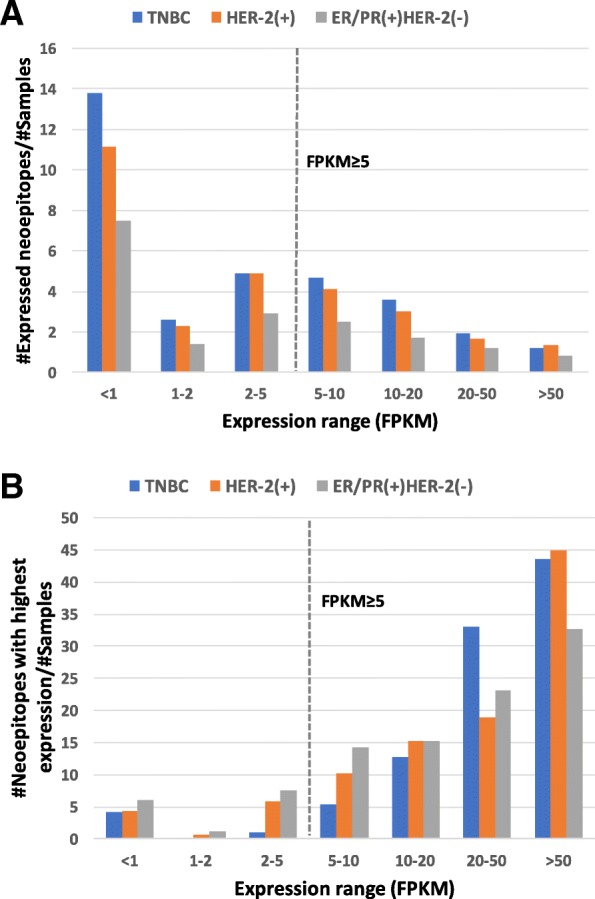
Expression analysis for the high affinity neoepitopes (FPKM ≥5). (**a**) The number of the expressed neoepitopes (normalized by total number of samples in each breast cancer subtype) is shown for each FPKM range. 35% (6098/17518) of the neoepitopes are expressed with an FPKM threshold of ≥5. (**b**) The number of neoepitopes (normalized by the number of samples in each breast cancer subtype) with the highest expressed neoepitope for each patient is shown. 87% (709/815) of patients have at least one potential binding epitope. < 1 includes neoepitopes with expression less than 1.0 FPKM (not including 1.0 FPKM), 1–2 includes neoepitopes with expression equal to or greater than one and less than 2 FPKM, and so on, for all categories

Using a threshold of expression less than 5 FPKM, two thirds (~ 65%) of the neoepitopes are not expressed (TNBC = 65.2%, HER-2(+) = 64.4%, ER/PR(+)HER-2(−) = 65.5%; Fig. 4a), and are thus considered not expressed. For the three subtypes, approximately half of the expressed neoepitopes have an expression value less than 2 FPKM (TNBC = 50.3%, HER-2(+) = 47.2%, ER/PR(+)HER-2(−) = 49.4%; Additional file 9: Figure S7A). Despite most neoepitopes not being expressed, we find that 87% (709/815) of patients have at least one expressed neoepitope (Fig. 4b), with a threshold of expression less than 5 FPKM. Similarly 93.5% (762/815) of patients have at least one expressed neoepitope with FPKM≥2: TNBC (*n* = 90/94, 96%); HER-2(+) (*n* = 131/138, 95%); and ER/PR(+)HER-2(−) (*n* = 541/583, 93%; Additional file 9: Figure S7B).

We also looked at the number of neoepitopes predicted for each HLA class I allele in the TCGA samples. The total number of neoantigens for each allele, as well as for each breast cancer subtype, are shown in Additional file 10: Table S3. We notice that different alleles retrieve different proportion of neoepitopes in the breast cancer subtypes. Overall, patients with the ER/PR(+)HER-2(−) subtype has a higher proportion of HLA-A binding alleles, while patients with the HER-2(+) and the TNBC subtypes have a higher proportion of HLA-B binding alleles (Additional file 11: Figure S8).

### Association of total mutation burden with clinical outcome

Mutation burden has been associated with clinical outcome in colorectal and ER(+) breast cancer patients [51, 52]. Here, we first compared the clinical outcome between high and low mutation burden groups. We defined the top quartile of mutation burden as high and the bottom quartile as low. No significant difference of disease-free survival was observed between high and low mutation burden groups in any subtype of breast cancer (Additional file 12: Figure S9). However, we noticed low mutation burden was associated with improved overall survival in the HER-2(+) subtype (*p* = 0.008; Additional file 12: Figure S9B). There were thirty-three patients in the low mutation burden group and thirty-seven subjects in the high mutation burden group; five patients died in the high mutation burden group, compared to none in the low mutation burden group.

We next compared the clinical outcome between high and low neoepitope load in the three subtypes of breast cancer. Neoepitope load was not associated with disease-free survival or overall survival (Additional file 13: Figure S10). In this study, we observed tumors that presented a higher neoepitope load (NEL) than the matched TMB (tumor mutation burden), indicative of neoepitopes that bind to multiple HLAs with high affinity and are expressed. There were more samples with NEL > TMB in TNBC (40%) than ER/PR(+)HER-2(−) (27%) and HER-2(+) (27%). We did not observe any association between high or low NEL/TMB ratio and prognosis (either disease-free survival or overall survival) across the breast cancer subtypes (Additional file 14: Figure S11).

## Discussion

We developed a pipeline to rapidly predict neoepitopes, EpitopeHunter (Fig. 1), and applied it to the TCGA breast cancer exome and expression data to characterize the mutational landscape of somatic mutations and predict clinically-relevant neoepitopes. Our pipeline for neoepitope prediction uses the advanced method (IEDB) for epitope binding affinity prediction, which selects the best method out of a few methods [40], SMM [41], NetMHCpan [42], Comblib [43] for a given MHC molecule. Other publically available pipelines [36, 37], on the other hand, use only one method to predict binding affinity. Using efficient and parallel computational resources, the pipeline can be used in clinical settings to provide a list of prioritized neoantigens that can be tested in vitro to find potential vaccine candidates. We confirmed that, of the three subtypes of cancer, the TNBC subtype has both the highest median mutational burden and highest median neoepitope load. Further, by incorporating analysis of expression, we found that approximately half of predicted neoepitopes are not expressed at the RNA level, suggesting that RNA expression data is needed to predict neoepitope vaccine candidates, as the majority of neoepitopes predicted from exome sequencing alone will not result in effective tumor targeting. That said, as many as 93.5% of breast cancer patients in this study have at least one expressed neoepitope that could serve as a candidate for a vaccine. Our analysis finds that overall neoepitope load is not associated with either disease-free survival, nor overall survival (Additional file 13: Figure S10). In contrast, for HER-2(+) only, we find that overall survival is higher in patients with a lower mutational burden. We hypothesize that the lack of correlation between mutational burden or neoantigen load and survival may be due to several factors, including different treatments among the patients in each class, or potentially that the magnitude of mutation in a tumor isn’t sufficient to predict survival, but rather, that it may be important to use additional criterion to identify the types of mutations and neoantigens that are associated with survival outcomes. Thus, it may be more important to prioritize and identify any high-affinity and expressed neoepitopes for vaccine development, rather than being able to identify many candidates. We also find that 9-mer neoepitopes are most often predicted to be the highest affinity binders across all breast cancer subtypes, with most, but not all, high affinity binding neoepitopes being expressed at least 2 FPKM (Fragments per Kilobase per transcript per Million mapped reads). Rooney et al. [53], identified high mutational burden in breast cancers, that we recapitulate. We extend on this by performing our own variant calling, annotation and high affinity binding neoepitope prediction of cancer specific variants for the three breast cancer subtypes (ER/PR(+)HER-2(−), HER-2(+), and TNBC), and found that TNBC has higher mutational burden than other subtypes. Our findings suggest that most breast cancers, especially TNBC, have strong candidate neoepitopes that may serve as targets for personalized vaccines, and identify patients who may benefit from checkpoint treatment.

The pioneering study of neoepitope prediction of breast cancer only includes neoepitopes restricted to HLA0201 [23], and reported about 10 neoepitopes per sample. Another study, including 760 breast cancer samples from the TCGA dataset, reported the average mutational burden being 52.3 (1–393) and predicted the neoepitopes (9mers and 10mers) binding to each patient’s imputed HLA alleles (< 500 nM), reporting an average of 9.6 neoepitopes per sample (0–64) [53]. An average of 679 and 449 mutations per sample were reported in the study of melanoma and lung cancer, yielding a higher number of mean neoepitopes per sample, 63 and 46 per sample, respectively [21]. Generally, 50% of non-silent mutations were found with ≥1 predicted neo-epitope across cancer types [53].

Checkpoint blockade therapies targeting the immune microenvironment have demonstrated clinical activity in multiple tumors [6, 54, 55], including TNBC [28, 56]. The response rate ranges from 20 to 40% even in sensitive tumors, such as melanoma and NSCLC [57]. The response rate of checkpoint block might be improved when combined with a targeted neoantigen vaccine.

In this study, we only analyzed single nucleotide variant for HLA class I restricted neoepitope prediction. Giannakis et al. has reported that frameshifts generate larger proportion of neoantigens than SNVs [58], and more neoantigen specific CD + 4 T cells have been identified after immunotherapy [19, 59], which indicates the broad potential targets in breast cancer. In the future, we can extend EpitopeHunter to include insertions and deletions, in addition to point mutations.

## Conclusions

Therapeutic tumor vaccination and CAR-T therapy have been tested in both preclinical and early phase clinical trials [19, 29, 60–63]. Two recent publications in melanoma evaluated mutation-specific personalized vaccination [18, 19], and multiple other trials are ongoing. Sustained progression free intervals have been observed in a subset of patients with vaccine alone or combined with a PD-1 inhibitor [64]. However, neo-epitope specific vaccination remains a manufacturing challenge due to the limited overlap of neoepitopes between patients, and identification of the dominant neoepitopes within tumors with multiple potential targets. Our analysis of the neoepitope landscape across subtypes of breast cancer – notably that over 90% of patients have at least one expressed neoepitope, provides a strong rationale for the development of tumor vaccine trials for breast cancer.

## Additional files

Additional file 1: **Table S1.** Number of patients for breast cancer subtype in the TCGA dataset according to IHC status. Sample sizes of the initial dataset, and for samples that were able to be included at each stage (mutation burden calling, neoepitope load estimation, and then gene expression analysis), are reported for each of the three breast cancer subtype categories (ER/PR(+)HER-2(−), HER-2(+), TNBC) (PDF 56 kb)

Additional file 2: **Table S2.** Patient and tumor characteristics. Demographic data for patients and clinical measurements for tumors are included in the table below for each category of breast cancer (PDF 58 kb)

Additional file 3: **Figure S1.** Frequently mutated genes in TCGA breast cancer data. Here we show the mo for: (A) ER/PR(+)HER-2(−) breast cancers, (B) HER-2(+) breast cancers, and (C) TNBC. The % Mutant indicates the percentage of samples with a mutation in the annotated particular gene (PDF 179 kb)

Additional file 4: **Figure S2.** Comparison of mutations called by the current protocol versus cBioportal **(**http://www.cbioportal.org). The total number of mutations called by the current protocol (in blue) is comparable to the mutations called from cBioportal (in orange) across all three classifications of breast cancer subtype (PDF 425 kb)

Additional file 5: **Figure S3.** Correlation of number of potential binding neoepitopes with number of nonsynonymous mutations. The number of potential binding neoepitopes (IEDB score ≤ 500) are highly correlated with the number of nonsynonymous mutations for all three subtypes of breast cancer. In all the plots, a linear regression model is used to fit the data; the fitted line is shown in red and 95% CIs are shown as grey shaded area around the line (PDF 268 kb)

Additional file 6: **Figure S4.** Length of potential binding neoepitopes in breast cancer. The number of potential binding neoepitopes (IEDB ≤500) are plotted against peptide sizes (8, 9, 10, 11 mers). 4% of the predicted neoepitopes are 8-mers, 57% of the predicted neoepitopes are 9-mer, 33% of the predicted neoepitopes are 10-mers and 6% are 11mers (PDF 101 kb)

Additional file 7: **Figure S5.** Expressed neoepitopes (FPKM≥5) in subtypes of breast cancer. The range of expressed neoepitopes (with FPKM≥5) is highest for the TNBC, followed by HER-2(+); and lowest for the ER/PR(+)HER-2(−) subtype of breast cancer. The median and range of the number of expressed neoepitopes are: 4 (0–131) in ER/PR(+)HER-2(−), 3 (0–82) in HER-2(+) and 8 (0–230) in TNBC. The number of samples in each case are: 583 (ER/PR(+)HER-2(−)), 138 (HER-2(+)), 92 (TNBC). Significant differences between reported FPKM values are computed pairwise for each breast cancer subtype using a Wilcox rank sum test, *** *P* < 0.001 (PDF 196 kb)

Additional file 8: **Figure S6.** Correlation of number of potential binding neoepitopes with number of expressed (FPKM≥2) neoepitopes. The number of potential binding neoepitopes (IEDB score ≤ 500) are highly correlated with the number of expressed neoepitopes (FPKM≥2) for all three subtypes of breast cancer. In all the plots a linear regression model is used to fit the data; the fitted line is shown in red and 95% CIs are shown in grey (PDF 255 kb)

Additional file 9: **Figure S7.** Expression analysis for the high affinity neoepitopes (FPKM ≥2). (A) The number of the expressed neoepitopes (normalized by total number of samples in each breast cancer subtype) is shown for each FPKM range. 51% (8927/17518) of predicted neoepitopes are expressed with an FPKM threshold of ≥2. (B) Number of neoepitopes (normalized by the number of samples in each breast cancer subtype) with the highest expressed neoepitope for each patient as shown. 93% (762/815) of patients have at least one potential binding epitope. < 1 includes neoepitopes with expression less than 1.0 FPKM (not including 1.0 FPKM), 1–2 includes neoepitopes with expression equal to or greater than one and less than 2 FPKM, and so on, for all categories (PDF 87 kb)

Additional file 10: **Table S3.** Number of neoepitopes predicted for HLA class I alleles in all the breast cancer samples as well as for the three subtypes. For each allele, we also list the proportion of neoepitopes for each subtype (PDF 54 kb)

Additional file 11: **Figure S8.** Distribution of neoepitopes binding to HLA class I alleles in the three subtypes of breast cancer. HLA-A and HLA-C binding alleles are seen in higher proportion in ER/PR(+)HER-2(−) subtype, while HLA-B binding alleles are in higher proportion in HER-2(+) and TNBC subtype. (PDF 240 kb)

Additional file 12: **Figure S9.** Kaplan-Meier estimates for high and low mutation burden. KM survival curves are shown for (A) disease-free survival and (B) overall survival between cases with high and low mutation (MB) burden, the upper and lower quartiles, in each subtype of breast cancer. Here we defined the top quartile of mutation burden as high and the bottom quartile as low (PDF 903 kb)

Additional file 13: **Figure S10.** Kaplan-Meier estimates by neoepitope load. KM curves of (A) disease-free survival and (B) overall survival between cases with high and low neoepitope load (NEL) in each subtype of breast cancer. High and low are defined as the upper and bottom quartile, respectively, for each breast cancer subtype (PDF 885 kb)

Additional file 14: **Figure S11.** Kaplan-Meier estimates based on mutation burden and neoepitope load. KS curves of (A) disease-free survival and (B) overall survival between cases with NeoEpitope Load (NEL) > Tumor Mutation Burden (TMB) and NEL < TMB in all three subtypes of breast cancer (PDF 234 kb)

## Abbreviations

FPKM: Fragments per Kilobase per transcript per Million mapped reads
NEL: Neoepitope load
SNV: Single Nucleotide Variant
TCGA: The Cancer Genome Atlas
TMB: Tumor mutational burden
TNBC: Triple Negative Breast Cancer
