# Supplementary material for: The neoepitope landscape of breast cancer: implications for immunotherapy
Source: BMC Cancer. 2019 Mar 4;19:200. doi: 10.1186/s12885-019-5402-1 (PMC6399957; doi:10.1186/s12885-019-5402-1)
Supplement: Supplementary file 1 — Table S1. Number of patients for breast cancer subtype in the TCGA dataset according to IHC status. Sample sizes of the initial dataset, and for samples that were able to be included at each stage (mutation burden calling, neoepitope load estimation, and then gene expression analysis), are reported for each of the three breast cancer subtype categories (ER/PR(+)HER-2(−), HER-2(+), TNBC) (PDF 56 kb) [file 12885_2019_5402_MOESM1_ESM.pdf]

Table S1. Number of patients for breast cancer subtype in the TCGA dataset according to IHC status. Sample sizes of the initial dataset, and for samples that were able to be included at each stage (mutation burden calling, neoepitope load estimation, and then gene expression analysis), are reported for each of the three breast cancer subtype categories (ER/PR(+)HER-2(-), HER-2(+), TNBC).

**Breast cancer subtype**	**Sample size**		
	**Mutation burden**	**Neoepitope load**	**Expression data**
**ER/PR(+)HER-2(-)**<br>ER(+) only, ER(+)/PR(+), PR(+) only, HER-2(-) for all	630	586	583
**HER-2(+)**<br>HER-2(+) only, ER(+)/PR(+)/HER-2(+), ER(+)/HER-2(+), PR(+)/HER-2(+)	141	138	138
**TNBC**<br>ER(-)/PR(-)/HER-2(-)	99	93	92
**Total**	870	817	721
