# Supplementary material for: The neoepitope landscape of breast cancer: implications for immunotherapy
Source: BMC Cancer. 2019 Mar 4;19:200. doi: 10.1186/s12885-019-5402-1 (PMC6399957; doi:10.1186/s12885-019-5402-1)
Supplement: Supplementary file 2 — Table S2. Patient and tumor characteristics. Demographic data for patients and clinical measurements for tumors are included in the table below for each category of breast cancer (PDF 58 kb) [file 12885_2019_5402_MOESM2_ESM.pdf]

Table S2. Patient and tumor characteristics. Demographic data for patients and clinical measurements for tumors are included in the table below for each category of breast cancer.

Characteristics	ER/PR(+)HER-2(-)		HER-2(+)		TNBC		Total
	n	%	n	%	n	%	
**Age (years)**							
**<50**	167	26.5	35	25.0	37	37	
**≥50**	462	73.3	106	75.2	62	63	
**NA**	1	0.1	0	0	0	0	
**Tumor size (cm)**							
**T1**	166	26.3	27	19.1	22	23	
**T2**	360	57.1	94	67.7	65	65	
**T3**	86	13.7	14	10.0	10	10	
**T4**	17	2.6	6	4.3	2	2.0	
**NA**	1	0.1	0	0	0	0	
**Lymph node status**							
**N0**	281	44.6	59	41.8	67	68	
**N1**	217	34.4	47	33.3	21	21	
**N2**	70	11.1	20	14.2	7	7	
**N3**	48	7.6	14	10.0	4	4	
**NX**	14	2.3	1	0.7	0	0	
**Tumor Stage**							
**Stage I**	108	17.2	16	11.3	17	17	
**Stage II**	349	55.4	83	58.9	65	65	
**Stage III**	146	23.2	37	26.2	14	14	
**Stage IV**	12	2.0	3	2.1	2	2	
**NA**	15	2.4	2	1.4	2	2	
Total	*630*	*72.4*	*141*	*16.2*	*99*	*11.4*	*870*
