# Supplementary material for: The neoepitope landscape of breast cancer: implications for immunotherapy
Source: BMC Cancer. 2019 Mar 4;19:200. doi: 10.1186/s12885-019-5402-1 (PMC6399957; doi:10.1186/s12885-019-5402-1)
Supplement: Supplementary file 3 — Figure S1. Frequently mutated genes in TCGA breast cancer data. Here we show the mo for: (A) ER/PR(+)HER-2(−) breast cancers, (B) HER-2(+) breast cancers, and (C) TNBC. The % Mutant indicates the percentage of samples with a mutation in the annotated particular gene (PDF 179 kb) [file 12885_2019_5402_MOESM3_ESM.pdf]

Figure S1. Frequently mutated genes in TCGA breast cancer data. Here we show the most mutated genes for: (A) ER/PR(+)HER-2(-) breast cancers, (B) HER-2(+) breast cancers, and (C) TNBC. The % Mutant indicates the percentage of samples with a mutation in the annotated particular gene.

A.

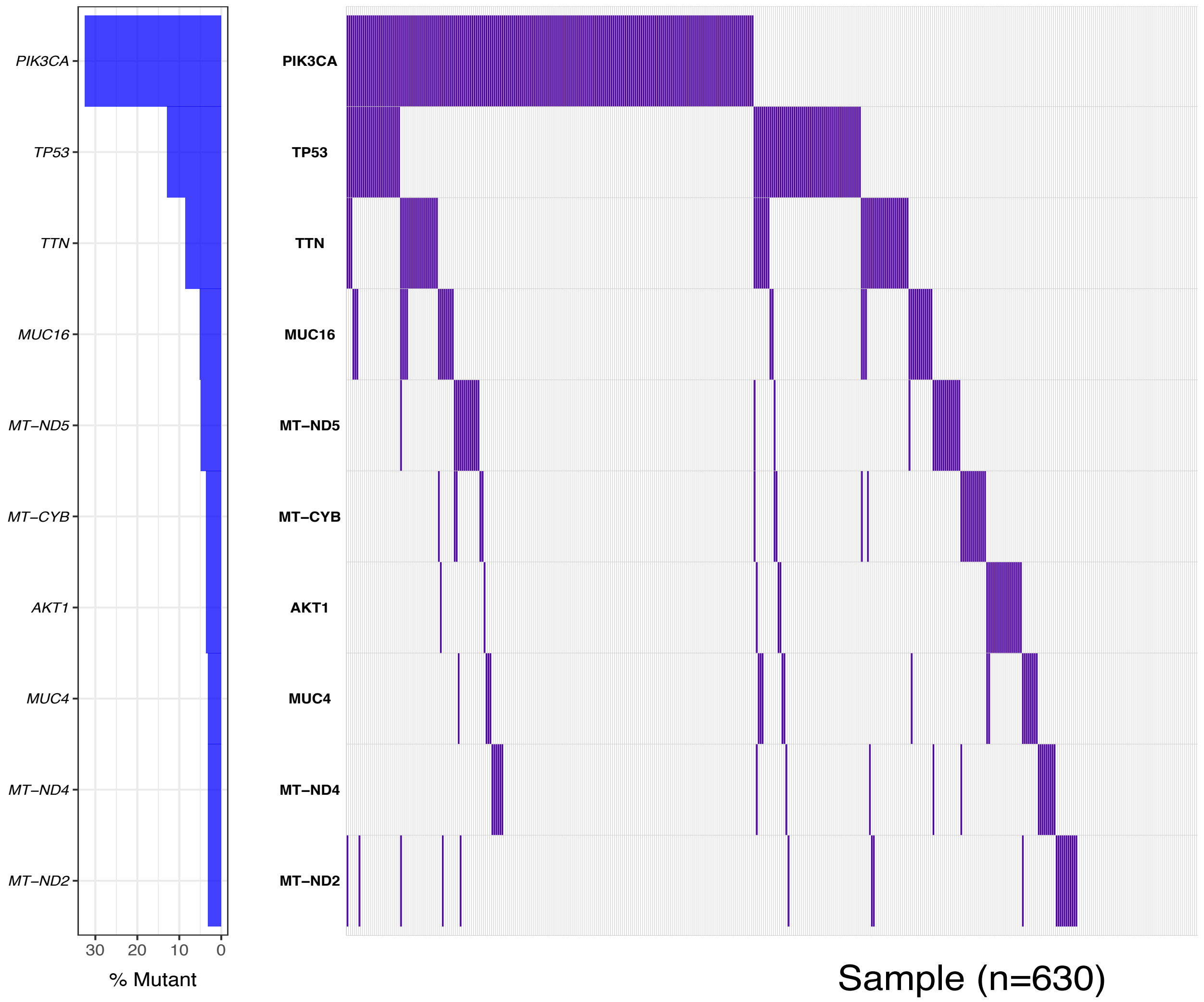

B.

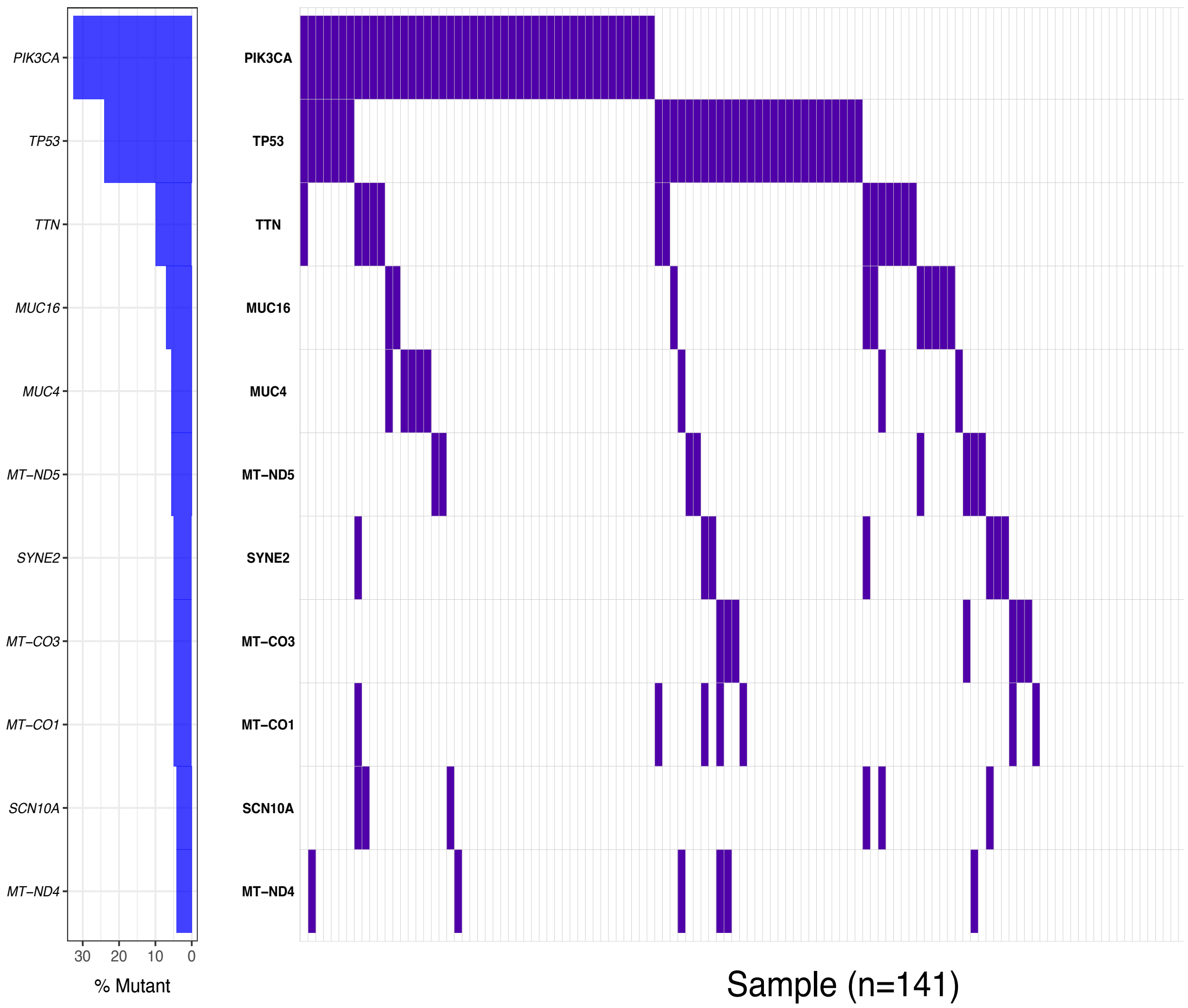

PIK3CA
TP53
TTN
MUC16
MUC4
MT−ND5
SYNE2
MT−CO3
MT−CO1
SCN10A
MT−ND4
30
20
10
0
% Mutant
PIK3CA
TP53
TTN
MUC16
MUC4
MT−ND5
SYNE2
MT−CO3
MT−CO1
SCN10A
MT−ND4
Sample (n=141)

C.

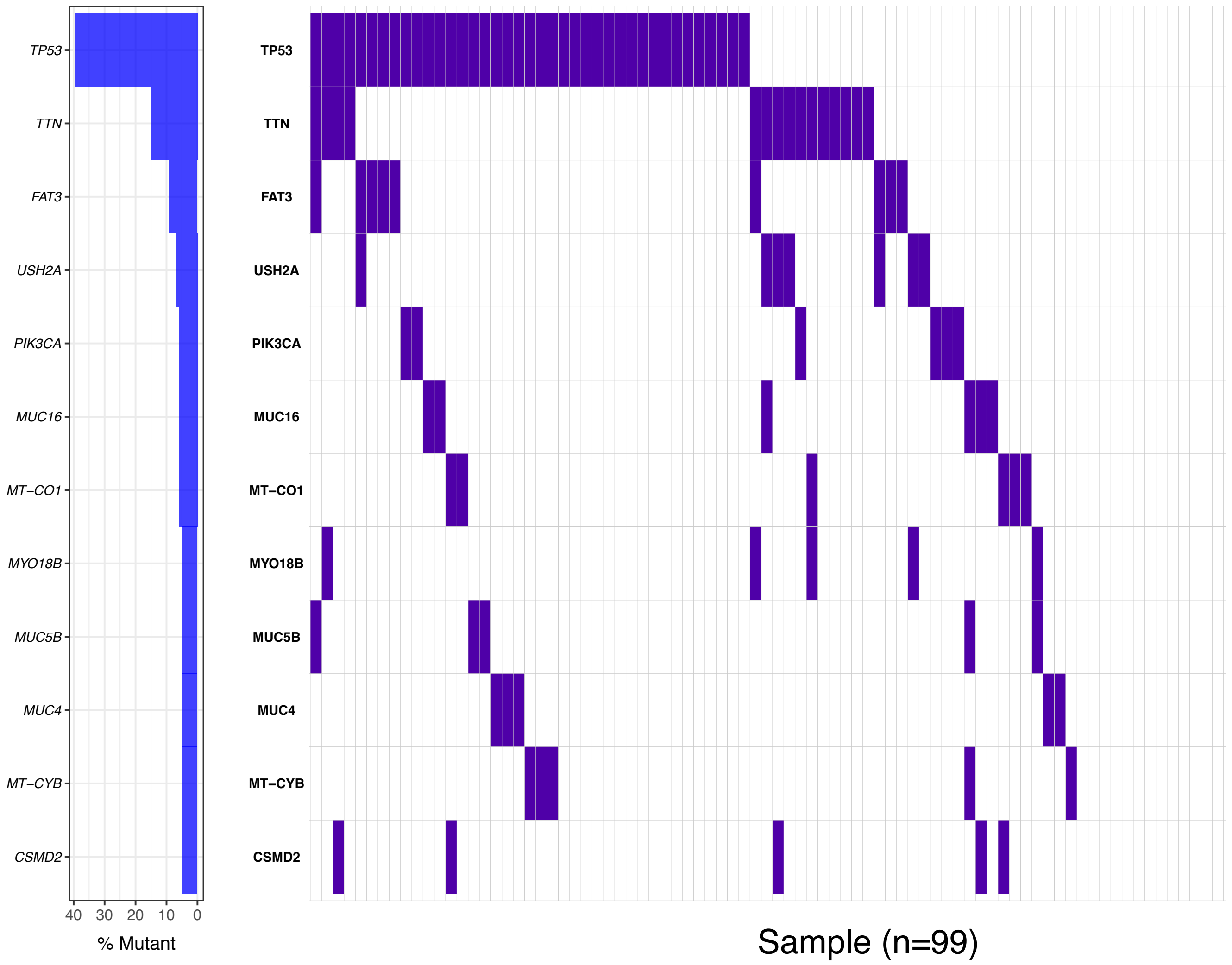
TP53
TTN
FAT3
USH2A
PIK3CA
MUC16
MT−CO1
MYO18B
MUC5B
MUC4
MT−CYB
CSMD2
40 30 20 10 0
% Mutant
Sample (n=99)
