# Supplementary material for: The neoepitope landscape of breast cancer: implications for immunotherapy
Source: BMC Cancer. 2019 Mar 4;19:200. doi: 10.1186/s12885-019-5402-1 (PMC6399957; doi:10.1186/s12885-019-5402-1)

Figure S2. Comparison of mutations called by the current protocol versus cBioportal (http://www.cbioportal.org). The total number of mutations called by the current protocol (in blue) is comparable to the mutations called from cBioportal (in orange) across all three classifications of breast cancer subtype.

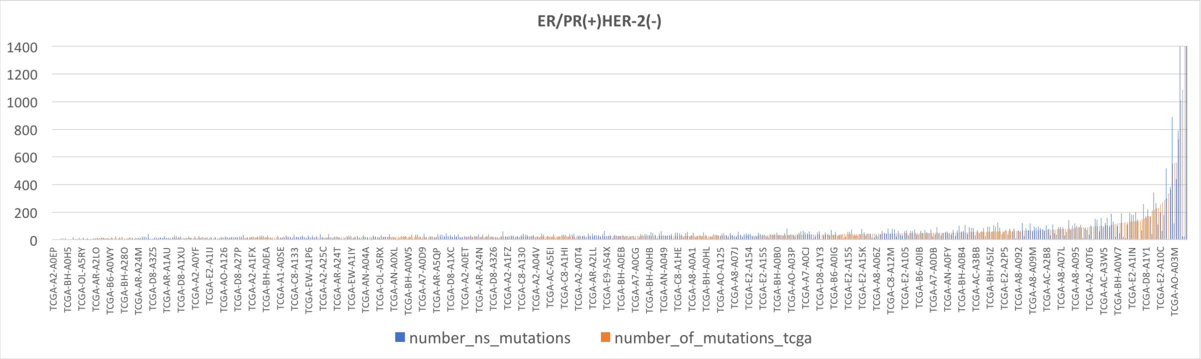

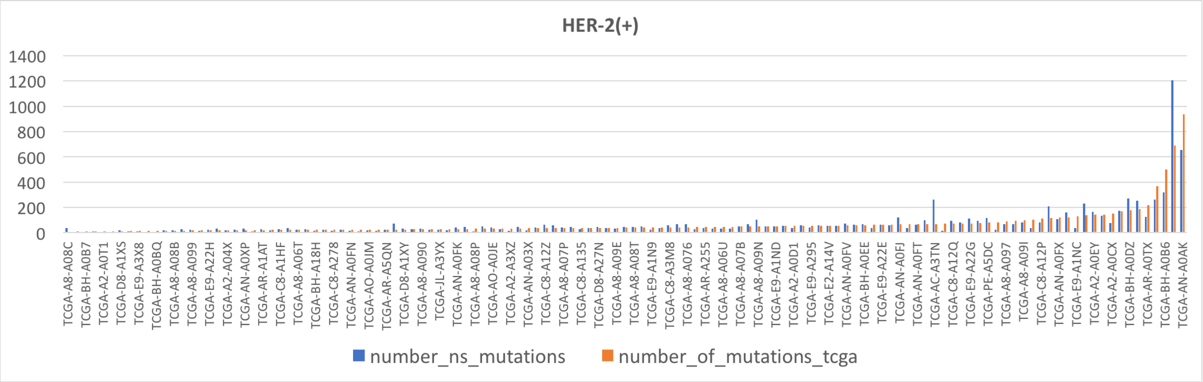

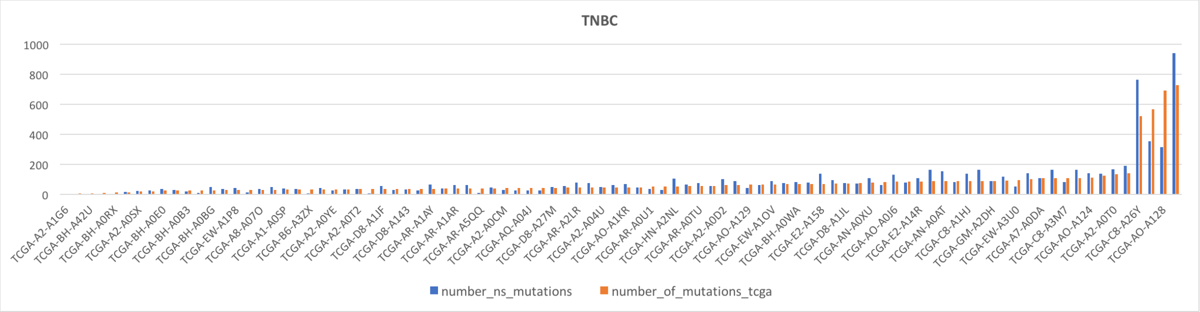

Supplement: Supplementary file 4 — Figure S2. Comparison of mutations called by the current protocol versus cBioportal (http://www.cbioportal.org). The total number of mutations called by the current protocol (in blue) is comparable to the mutations called from cBioportal (in orange) across all three classifications of breast cancer subtype (PDF 425 kb) [file 12885_2019_5402_MOESM4_ESM.pdf]
