# Supplementary material for: The neoepitope landscape of breast cancer: implications for immunotherapy
Source: BMC Cancer. 2019 Mar 4;19:200. doi: 10.1186/s12885-019-5402-1 (PMC6399957; doi:10.1186/s12885-019-5402-1)
Supplement: Supplementary file 5 — Figure S3. Correlation of number of potential binding neoepitopes with number of nonsynonymous mutations. The number of potential binding neoepitopes (IEDB score ≤ 500) are highly correlated with the number of nonsynonymous mutations for all three subtypes of breast cancer. In all the plots, a linear regression model is used to fit the data; the fitted line is shown in red and 95% CIs are shown as grey shaded area around the line (PDF 268 kb) [file 12885_2019_5402_MOESM5_ESM.pdf]

Figure S3. Correlation of number of potential binding neoepitopes with number of nonsynonymous mutations. The number of potential binding neoepitopes (IEDB score ≤ 500) are highly correlated with the number of nonsynonymous mutations for all three subtypes of breast cancer. In all the plots, a linear regression model is used to fit the data; the fitted line is shown in red and 95% CIs are shown as grey shaded area around the line.

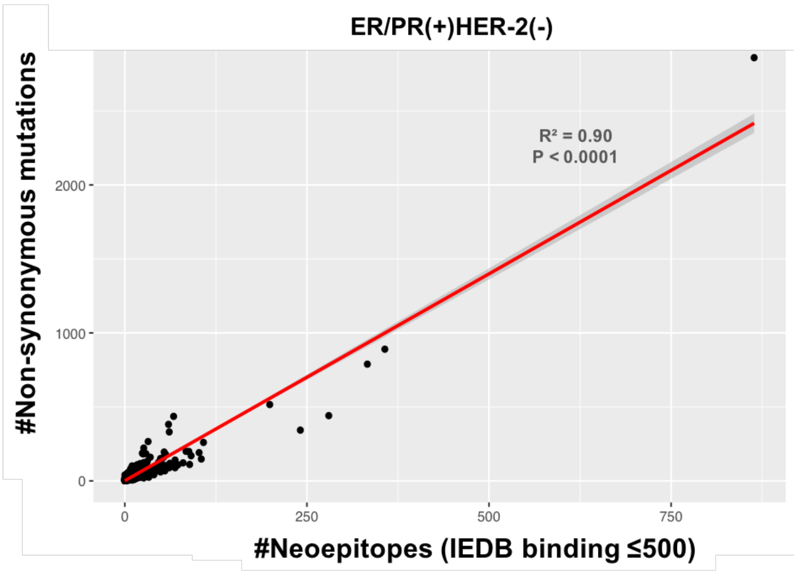

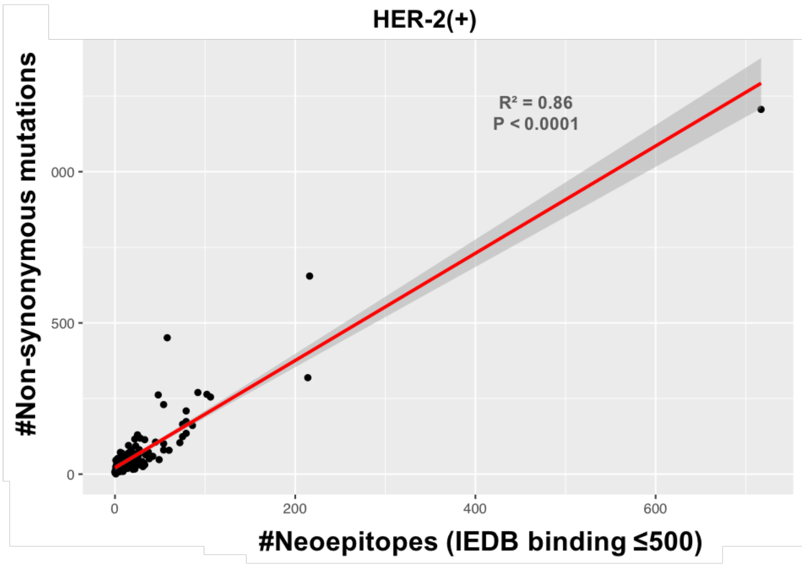

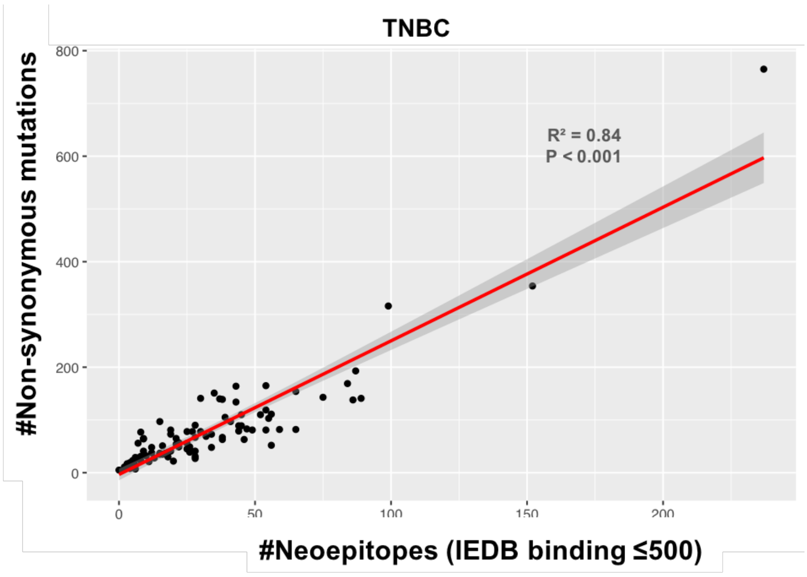

TNBC
#Non-synonymous mutations
#Neoepitopes (IEDB binding ≤500)
R² = 0.84
P < 0.001
800
600
400
200
0
0
50
100
150
200
