# Supplementary material for: The neoepitope landscape of breast cancer: implications for immunotherapy
Source: BMC Cancer. 2019 Mar 4;19:200. doi: 10.1186/s12885-019-5402-1 (PMC6399957; doi:10.1186/s12885-019-5402-1)

Figure S4. Length of potential binding neoepitopes in breast cancer. The number of potential binding neoepitopes (IEDB ≤ 500) are plotted against peptide sizes (8, 9, 10, 11 mers). 4% of the predicted neoepitopes are 8-mers, 57% of the predicted neoepitopes are 9-mer, 33% of the predicted neoepitopes are 10-mers and 6% are 11mers.

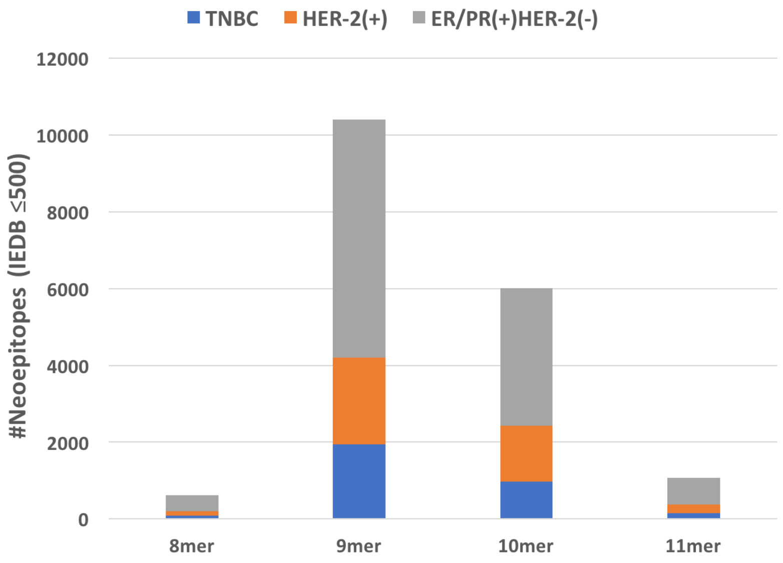

Supplement: Supplementary file 6 — Figure S4. Length of potential binding neoepitopes in breast cancer. The number of potential binding neoepitopes (IEDB ≤500) are plotted against peptide sizes (8, 9, 10, 11 mers). 4% of the predicted neoepitopes are 8-mers, 57% of the predicted neoepitopes are 9-mer, 33% of the predicted neoepitopes are 10-mers and 6% are 11mers (PDF 101 kb) [file 12885_2019_5402_MOESM6_ESM.pdf]
