# Supplementary material for: The neoepitope landscape of breast cancer: implications for immunotherapy
Source: BMC Cancer. 2019 Mar 4;19:200. doi: 10.1186/s12885-019-5402-1 (PMC6399957; doi:10.1186/s12885-019-5402-1)

Figure S5: Expressed neoepitopes (FPKM≥2) in subtypes of breast cancer. The range of expressed neoepitopes (with FPKM≥2) is highest for the TNBC, followed by HER-2(+); and lowest for the ER/PR(+)HER-2(-) subtype of breast cancer. The median and range of the number of expressed neoepitopes are: 4 (0-131) in ER/PR(+)HER-2(-), 6 (0-338) in HER-2(+) and 11 (0-381) in TNBC. The number of samples in each case are: 583 (ER/PR(+)HER-2(-)), 138 (HER-2(+)), 92 (TNBC). Significant differences between reported FPKM values are computed pairwise for each breast cancer subtype using a Wilcox rank sum test, *** $P < 0.001$.

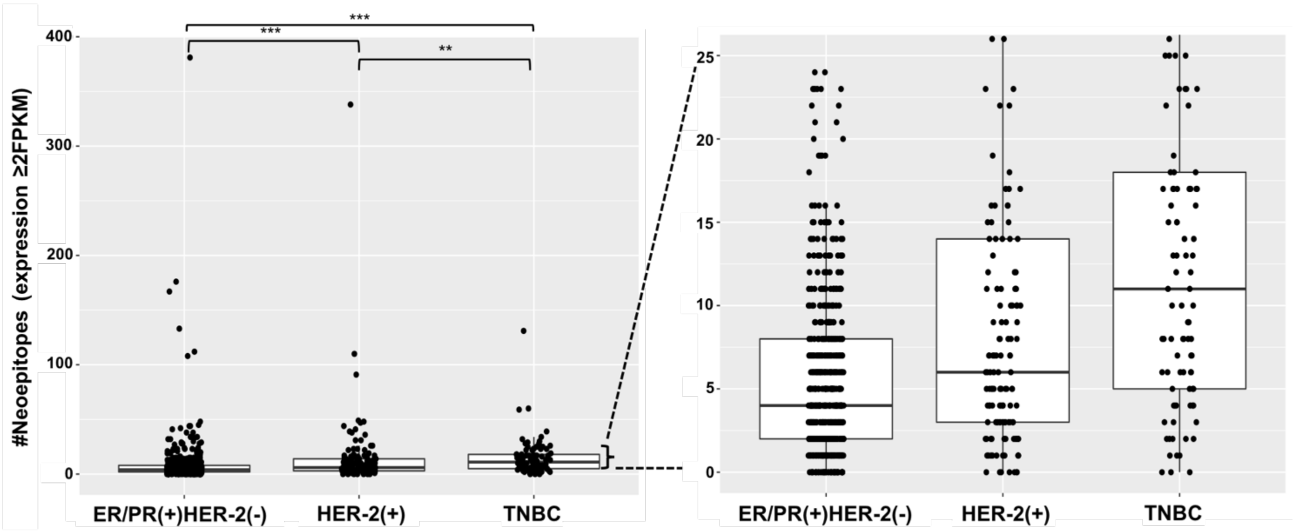

Supplement: Supplementary file 7 — Figure S5. Expressed neoepitopes (FPKM≥5) in subtypes of breast cancer. The range of expressed neoepitopes (with FPKM≥5) is highest for the TNBC, followed by HER-2(+); and lowest for the ER/PR(+)HER-2(−) subtype of breast cancer. The median and range of the number of expressed neoepitopes are: 4 (0–131) in ER/PR(+)HER-2(−), 3 (0–82) in HER-2(+) and 8 (0–230) in TNBC. The number of samples in each case are: 583 (ER/PR(+)HER-2(−)), 138 (HER-2(+)), 92 (TNBC). Significant differences between reported FPKM values are computed pairwise for each breast cancer subtype using a Wilcox rank sum test, *** P < 0.001 (PDF 196 kb) [file 12885_2019_5402_MOESM7_ESM.pdf]
