# Supplementary material for: The neoepitope landscape of breast cancer: implications for immunotherapy
Source: BMC Cancer. 2019 Mar 4;19:200. doi: 10.1186/s12885-019-5402-1 (PMC6399957; doi:10.1186/s12885-019-5402-1)
Supplement: Supplementary file 8 — Figure S6. Correlation of number of potential binding neoepitopes with number of expressed (FPKM≥2) neoepitopes. The number of potential binding neoepitopes (IEDB score ≤ 500) are highly correlated with the number of expressed neoepitopes (FPKM≥2) for all three subtypes of breast cancer. In all the plots a linear regression model is used to fit the data; the fitted line is shown in red and 95% CIs are shown in grey (PDF 255 kb) [file 12885_2019_5402_MOESM8_ESM.pdf]

Figure S6. Correlation of number of potential binding neoepitopes with number of expressed (FPKM≥2) neoepitopes. The number of potential binding neoepitopes (IEDB score ≤ 500) are highly correlated with the number of expressed neoepitopes (FPKM≥2) for all three subtypes of breast cancer. In all the plots a linear regression model is used to fit the data; the fitted line is shown in red and 95% CIs are shown in grey.

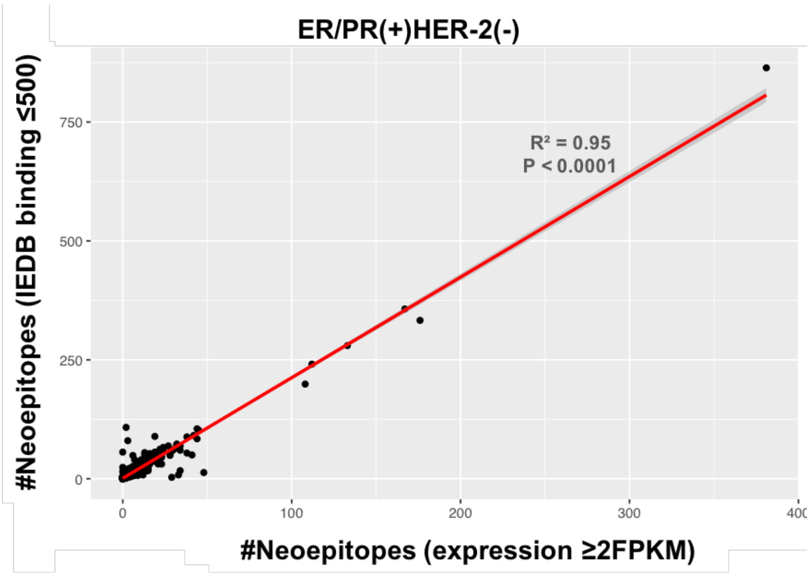

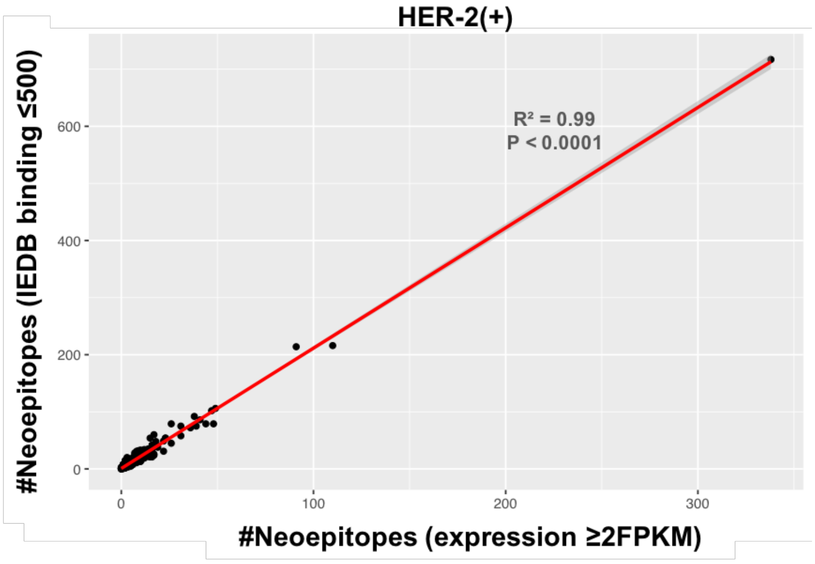

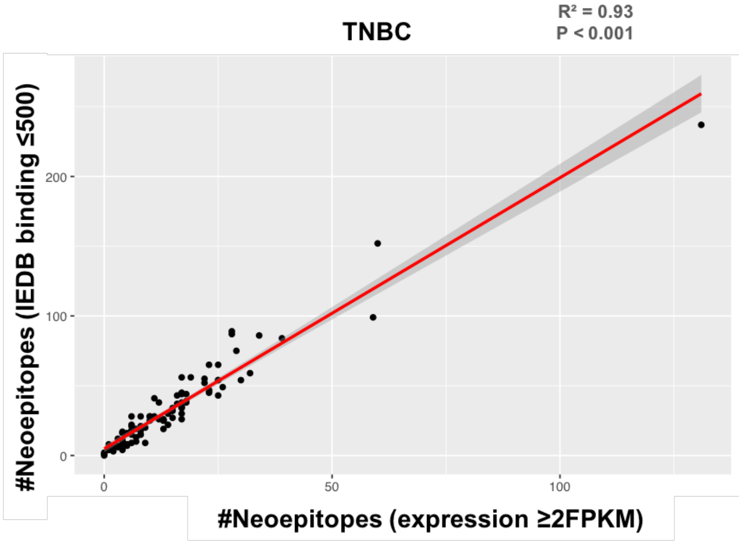

TNBC
R² = 0.93
P < 0.001
#Neoepitopes (IEDB binding ≤500)
200
100
0
0
50
100
#Neoepitopes (expression ≥2FPKM)
