# Supplementary material for: The neoepitope landscape of breast cancer: implications for immunotherapy
Source: BMC Cancer. 2019 Mar 4;19:200. doi: 10.1186/s12885-019-5402-1 (PMC6399957; doi:10.1186/s12885-019-5402-1)

Figure S7. Expression analysis for the high affinity neoepitopes (FPKM ≥2). (A) The number of the expressed neoepitopes (normalized by total number of samples in each breast cancer subtype) is shown for each FPKM range. 51% (8927/17518) of predicted neoepitopes are expressed with an FPKM threshold of ≥2. (B) Number of neoepitopes (normalized by the number of samples in each breast cancer subtype) with the highest expressed neoepitope for each patient as shown. 93% (762/815) of patients have at least one potential binding epitope. <1 includes neoepitopes with expression less than 1.0 FPKM (not including 1.0 FPKM), 1-2 includes neoepitopes with expression equal to or greater than one and less than 2 FPKM, and so on, for all categories.

(A)

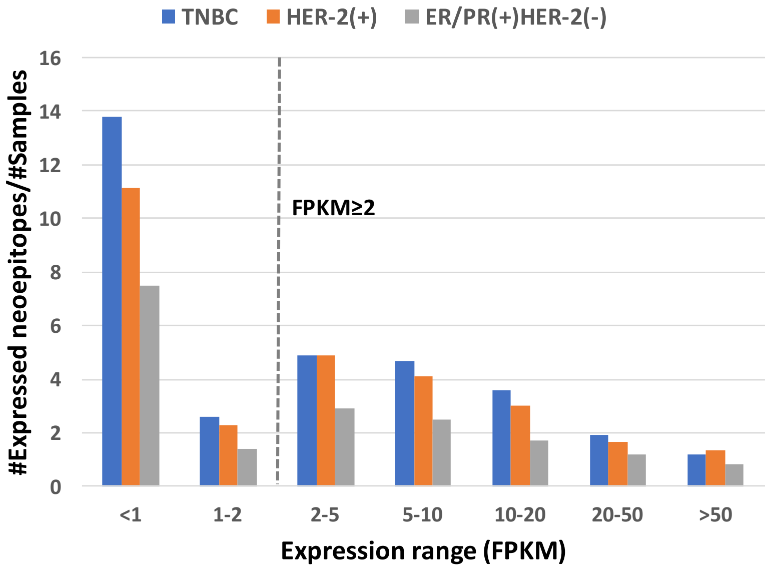

(B)

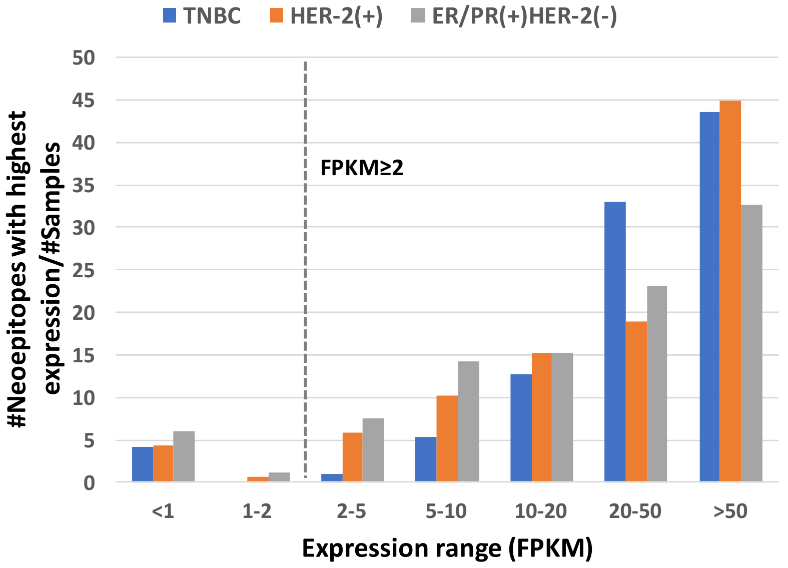

Supplement: Supplementary file 9 — Figure S7. Expression analysis for the high affinity neoepitopes (FPKM ≥2). (A) The number of the expressed neoepitopes (normalized by total number of samples in each breast cancer subtype) is shown for each FPKM range. 51% (8927/17518) of predicted neoepitopes are expressed with an FPKM threshold of ≥2. (B) Number of neoepitopes (normalized by the number of samples in each breast cancer subtype) with the highest expressed neoepitope for each patient as shown. 93% (762/815) of patients have at least one potential binding epitope. < 1 includes neoepitopes with expression less than 1.0 FPKM (not including 1.0 FPKM), 1–2 includes neoepitopes with expression equal to or greater than one and less than 2 FPKM, and so on, for all categories (PDF 87 kb) [file 12885_2019_5402_MOESM9_ESM.pdf]
