# Supplementary material for: The neoepitope landscape of breast cancer: implications for immunotherapy
Source: BMC Cancer. 2019 Mar 4;19:200. doi: 10.1186/s12885-019-5402-1 (PMC6399957; doi:10.1186/s12885-019-5402-1)
Supplement: Supplementary file 10 — Table S3. Number of neoepitopes predicted for HLA class I alleles in all the breast cancer samples as well as for the three subtypes. For each allele, we also list the proportion of neoepitopes for each subtype (PDF 54 kb) [file 12885_2019_5402_MOESM10_ESM.pdf]

Table S3: Number of neoepitopes predicted for HLA class I alleles in all the breast cancer samples as well as for the three subtypes. For each allele, we also list the proportion of neoepitopes for each subtype.

	#neoepitopes(prop of neoepitopes)			
Alleles	ER/PR(+)HER-2(-)	HER-2(+)	TNBC	Total
HLA-A*01:01	181(0.79)	33(0.14)	14(0.06)	228
HLA-A*02:01	368(0.55)	191(0.29)	111(0.17)	670
HLA-A*02:06	18(0.72)	3(0.12)	4(0.16)	25
HLA-A*03:01	299(0.58)	166(0.32)	54(0.1)	519
HLA-A*11:01	128(0.59)	32(0.15)	57(0.26)	217
HLA-A*23:01	24(0.56)	5(0.12)	14(0.33)	43
HLA-A*24:02	62(0.56)	30(0.27)	19(0.17)	111
HLA-A*25:01	1(0.33)	1(0.33)	1(0.33)	3
HLA-A*26:01	43(0.83)	3(0.06)	6(0.12)	52
HLA-A*29:02	21(0.66)	2(0.06)	9(0.28)	32
HLA-A*30:01	29(0.41)	16(0.23)	25(0.36)	70
HLA-A*30:02	13(0.68)	3(0.16)	3(0.16)	19
HLA-A*31:01	21(0.35)	13(0.22)	26(0.43)	60
HLA-A*32:01	15(0.52)	5(0.17)	9(0.31)	29
HLA-A*33:03	48(0.38)	17(0.13)	63(0.49)	128
HLA-A*68:01	102(0.58)	48(0.27)	25(0.14)	175
HLA-A*68:02	18(0.44)	3(0.07)	20(0.49)	41
HLA-A*74:01	1(0.5)	0(0)	1(0.5)	2
HLA-B*07:02	524(0.69)	150(0.2)	81(0.11)	755
HLA-B*08:01	101(0.67)	32(0.21)	17(0.11)	150
HLA-B*13:01	0(0)	1(1)	0(0)	1
HLA-B*13:02	0(0)	0(0)	1(1)	1
HLA-B*14:02	3(0.6)	0(0)	2(0.4)	5
HLA-B*15:01	140(0.68)	54(0.26)	12(0.06)	206
HLA-B*15:02	7(0.44)	2(0.13)	7(0.44)	16
HLA-B*15:25	17(0.52)	2(0.06)	14(0.42)	33
HLA-B*18:01	115(0.88)	6(0.05)	10(0.08)	131
HLA-B*27:02	1(0.03)	2(0.07)	26(0.9)	29
HLA-B*27:05	57(0.72)	22(0.28)	0(0)	79
HLA-B*35:01	74(0.53)	41(0.29)	24(0.17)	139
HLA-B*35:03	2(1)	0(0)	0(0)	2
HLA-B*39:01	11(0.37)	4(0.13)	15(0.5)	30
HLA-B*40:01	28(0.31)	50(0.56)	11(0.12)	89

HLA-B*40:02	45(0.69)	7(0.11)	13(0.2)	65
HLA-B*44:02	24(0.53)	17(0.38)	4(0.09)	45
HLA-B*44:03	30(0.79)	1(0.03)	7(0.18)	38
HLA-B*46:01	0(0)	0(0)	1(1)	1
HLA-B*48:01	0(0)	0(0)	1(1)	1
HLA-B*49:01	1(0.5)	1(0.5)	0(0)	2
HLA-B*50:01	0(0)	0(0)	1(1)	1
HLA-B*51:01	8(0.73)	1(0.09)	2(0.18)	11
HLA-B*52:01	0(0)	1(1)	0(0)	1
HLA-B*53:01	7(0.32)	0(0)	15(0.68)	22
HLA-B*55:01	1(0.14)	2(0.29)	4(0.57)	7
HLA-B*56:01	4(0.5)	1(0.13)	3(0.38)	8
HLA-B*57:01	10(0.23)	30(0.7)	3(0.07)	43
HLA-B*58:01	7(0.58)	4(0.33)	1(0.08)	12
HLA-C*01:02	1(1)	0(0)	0(0)	1
HLA-C*02:02	34(0.67)	17(0.33)	0(0)	51
HLA-C*02:09	1(0.17)	0(0)	5(0.83)	6
HLA-C*03:02	13(0.15)	20(0.23)	55(0.63)	88
HLA-C*03:03	81(0.64)	27(0.21)	18(0.14)	126
HLA-C*03:04	57(0.34)	73(0.43)	39(0.23)	169
HLA-C*05:01	24(0.51)	11(0.23)	12(0.26)	47
HLA-C*06:02	27(0.54)	16(0.32)	7(0.14)	50
HLA-C*07:01	93(0.78)	12(0.1)	14(0.12)	119
HLA-C*07:02	36(0.53)	18(0.26)	14(0.21)	68
HLA-C*08:01	6(0.43)	0(0)	8(0.57)	14
HLA-C*12:02	38(0.61)	24(0.39)	0(0)	62
HLA-C*12:03	83(0.73)	16(0.14)	14(0.12)	113
HLA-C*14:02	14(0.39)	9(0.25)	13(0.36)	36
HLA-C*15:02	17(0.47)	6(0.17)	13(0.36)	36
HLA-C*16:01	172(0.9)	2(0.01)	17(0.09)	191
HLA-C*17:01	1(0.33)	1(0.33)	1(0.33)	3
