# Supplementary material for: The neoepitope landscape of breast cancer: implications for immunotherapy
Source: BMC Cancer. 2019 Mar 4;19:200. doi: 10.1186/s12885-019-5402-1 (PMC6399957; doi:10.1186/s12885-019-5402-1)

Figure S8: Distribution of neoepitopes binding to HLA class I alleles in the three subtypes of breast cancer. HLA-A and HLA-C binding alleles are seen in higher proportion in ER/PR(+)HER-2(-) subtype, while HLA-B binding alleles are in higher proportion in HER-2(+) and TNBC subtype.

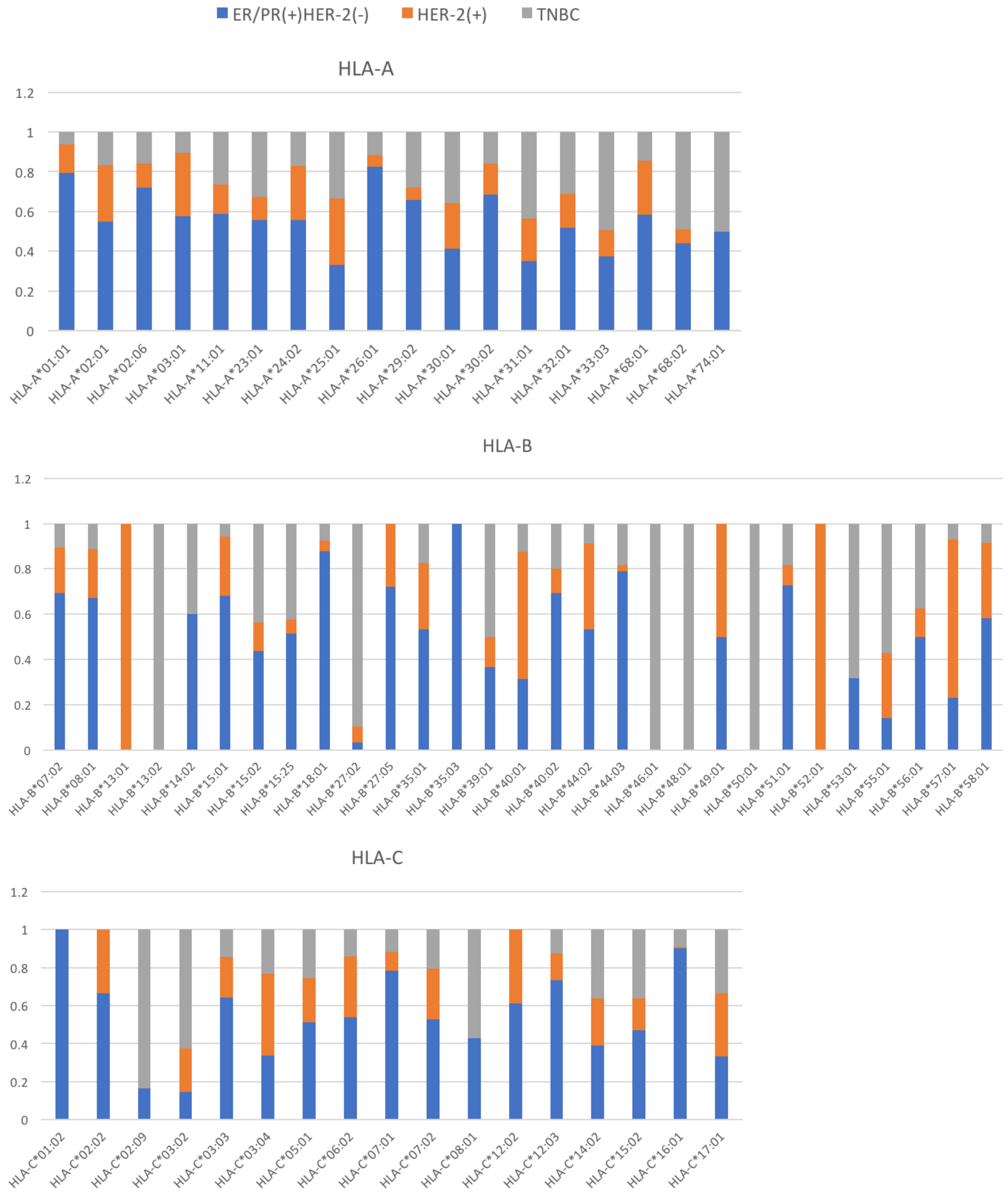

Supplement: Supplementary file 11 — Figure S8. Distribution of neoepitopes binding to HLA class I alleles in the three subtypes of breast cancer. HLA-A and HLA-C binding alleles are seen in higher proportion in ER/PR(+)HER-2(−) subtype, while HLA-B binding alleles are in higher proportion in HER-2(+) and TNBC subtype. (PDF 240 kb) [file 12885_2019_5402_MOESM11_ESM.pdf]
