# Supplementary material for: The neoepitope landscape of breast cancer: implications for immunotherapy
Source: BMC Cancer. 2019 Mar 4;19:200. doi: 10.1186/s12885-019-5402-1 (PMC6399957; doi:10.1186/s12885-019-5402-1)

Figure S9. Kaplan-Meier estimates for high and low mutation burden. KM survival curves are shown for (A) disease-free survival and (B) overall survival between cases with high and low mutation (MB) burden, the upper and lower quartiles, in each subtype of breast cancer. Here we defined the top quartile of mutation burden as high and the bottom quartile as low.

A.

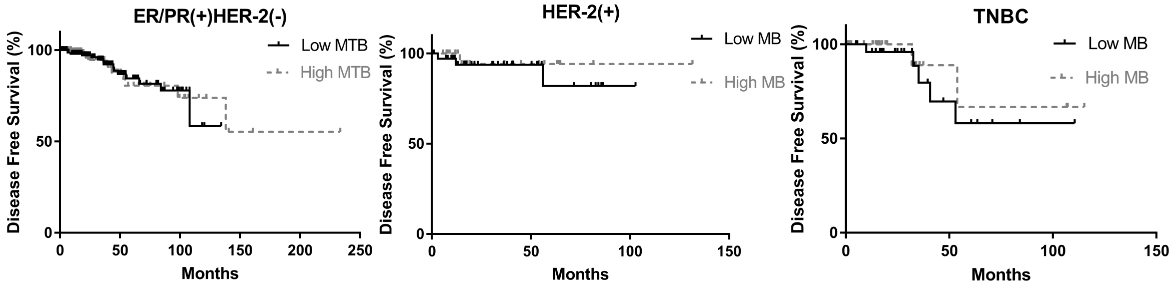

B.

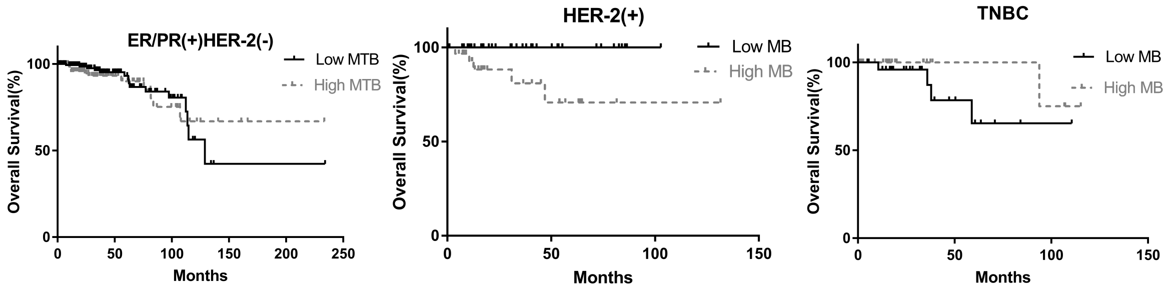

Supplement: Supplementary file 12 — Figure S9. Kaplan-Meier estimates for high and low mutation burden. KM survival curves are shown for (A) disease-free survival and (B) overall survival between cases with high and low mutation (MB) burden, the upper and lower quartiles, in each subtype of breast cancer. Here we defined the top quartile of mutation burden as high and the bottom quartile as low (PDF 903 kb) [file 12885_2019_5402_MOESM12_ESM.pdf]
