# Supplementary material for: The neoepitope landscape of breast cancer: implications for immunotherapy
Source: BMC Cancer. 2019 Mar 4;19:200. doi: 10.1186/s12885-019-5402-1 (PMC6399957; doi:10.1186/s12885-019-5402-1)

Figure S10. Kaplan-Meier estimates by neoepitope load. KM curves of (A) disease-free survival and (B) overall survival between cases with high and low neoepitope load (NEL) in each subtype of breast cancer. High and low are defined as the upper and bottom quartile, respectively, for each breast cancer subtype. Neoepitope load defined as FPKM>5.

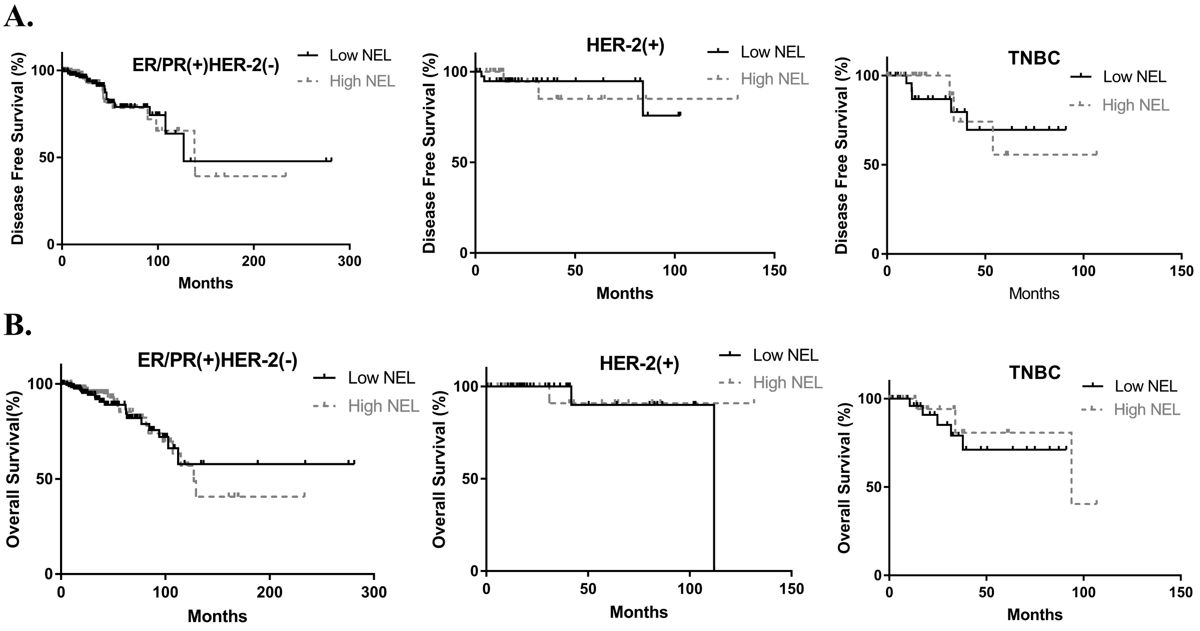

Supplement: Supplementary file 13 — Figure S10. Kaplan-Meier estimates by neoepitope load. KM curves of (A) disease-free survival and (B) overall survival between cases with high and low neoepitope load (NEL) in each subtype of breast cancer. High and low are defined as the upper and bottom quartile, respectively, for each breast cancer subtype (PDF 885 kb) [file 12885_2019_5402_MOESM13_ESM.pdf]
