# Supplementary material for: The neoepitope landscape of breast cancer: implications for immunotherapy
Source: BMC Cancer. 2019 Mar 4;19:200. doi: 10.1186/s12885-019-5402-1 (PMC6399957; doi:10.1186/s12885-019-5402-1)

Figure S11. Kaplan-Meier estimates based on mutation burden and total neoepitope load. KS curves of (A) disease-free survival and (B) overall survival between cases with total Neoepitope Load (NEL) > Tumor Mutation Burden (TMB) and NEL<TMB in all three subtypes of breast cancer.

A.

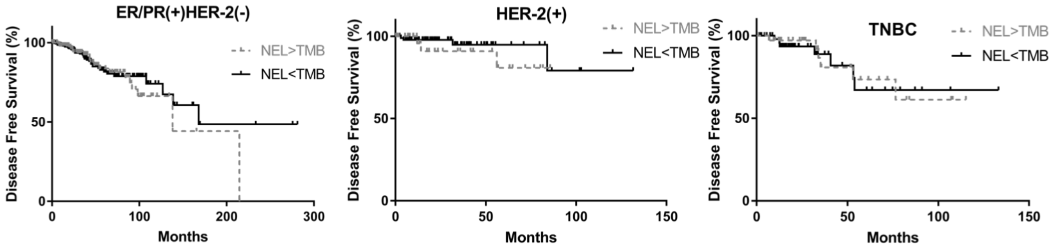

B.

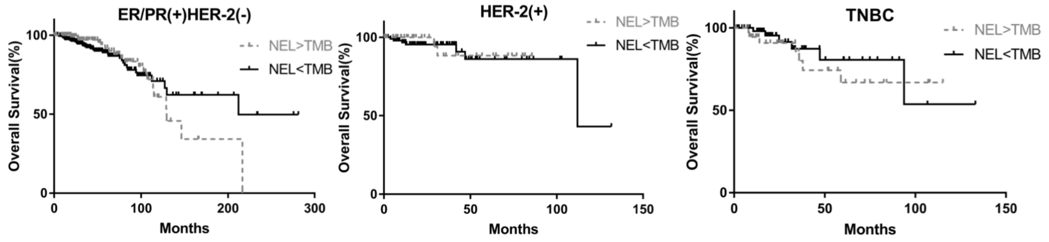

Supplement: Supplementary file 14 — Figure S11. Kaplan-Meier estimates based on mutation burden and neoepitope load. KS curves of (A) disease-free survival and (B) overall survival between cases with NeoEpitope Load (NEL) > Tumor Mutation Burden (TMB) and NEL < TMB in all three subtypes of breast cancer (PDF 234 kb) [file 12885_2019_5402_MOESM14_ESM.pdf]
